# Downregulation of JAM3 occurs in cholangiocarcinoma by hypermethylation: A potential molecular marker for diagnosis and prognosis

**DOI:** 10.1111/jcmm.18038

**Published:** 2023-12-20

**Authors:** Yi Shi, Xiao Feng, Ying Zhang, Ji Gao, Wei Bao, Jian‐dong Wang, Jian‐feng Bai

**Affiliations:** ^1^ Hepatobiliary Center The First Affiliated Hospital of Nanjing Medical University, Key Laboratory of Liver Transplantation, Chinese Academy of Medical Sciences, NHC Key Laboratory of Living Donor Liver Transplantation (Nanjing Medical University) Nanjing China; ^2^ Department of Pathology, Jiangsu Province Hospital of Chinese Medicine Affiliated Hospital of Nanjing University of Chinese Medicine Nanjing China; ^3^ Department of Pathology, the Affiliated Jinling Hospital Nanjing Medical University Nanjing China

**Keywords:** cholangiocarcinoma, hypermethylation, JAM3, junctional adhesion molecular 3, molecular marker

## Abstract

Junctional adhesion molecular 3 (JAM3) is downregulated by hypermethylation in cancers but is unclear in cholangiocarcinoma. The JAM3 expression level was checked in cholangiocarcinoma cell lines and tissues. Methylated JAM3 was detected in cell lines, tissues and plasma cell‐free DNAs (cfDNA). The roles of JAM3 in cholangiocarcinoma were studied by transfection of siRNA and pCMV3‐JAM3. The survival analysis was based on the Gene Set Cancer Analysis (GSCA) database. JAM3 was downregulated in HCCC‐9810 and HuCCT1 cell lines and tissues by hypermethylation. Methylated JAM3 was detected in cfDNAs with 53.3% sensitivity and 96.6% specificity. Transfection of pCMV3‐JAM3 into HCCC‐9810 and HuCCT1 induced apoptosis and suppressed cell proliferation, migration and invasion. The depletion of JAM3 in RBE cells using siRNA decreased apoptosis and increased cell proliferation, migration and invasion. Hypermethylation of JAM3 was associated with tumour differentiation, metastasis and TNM stage. Downregulation and hypermethylation of JAM3 were related to poor progression‐free survival. Junctional adhesion molecular 3 may function as a tumour suppressor in cholangiocarcinoma. Methylated JAM3 DNA may represent a non‐invasive molecular marker for the early detection of cholangiocarcinoma and prognosis.

## 1 INTRODUCTION

Cholangiocarcinoma (CCA) is a rare and aggressive tumour that arises from the biliary tree and is commonly classified according to its anatomic location^1^, ^2^, ^3^: intrahepatic (iCCA), perihilar (pCCA), or distal (dCCA) cholangiocarcinoma according to its anatomic location of origin.^4^ Perihilar CCA and dCCA account for approximately 80% of all CCAs diagnosed in the USA, while iCCA accounts for the remaining 20%.^4^ The overall incidence of CCA has increased progressively worldwide over the past four decades. Cholangiocarcinoma comprises 3% of all gastrointestinal tumours and is the second most common liver cancer, accounting for 5%–20% of all liver malignancies.^1^, ^2^ Most patients with CCA, due to its aggressiveness and silent clinical characteristics, have advanced‐stage disease at diagnosis. Indeed, most patients with early‐stage CCA are asymptomatic.^4^ Cholangiocarcinoma is characterised by early nodal and vascular invasion and carries a dismal prognosis.^1^ The mean 5‐year survival rate of CCA is <5% for late‐stage patients.^5^ Surgical resection is currently the most commonly used treatment but has unsatisfactory outcomes due to high recurrence rates and metastasis. Early diagnosis of CCA remains a challenge owing to its asymptomatic presentation at the early stage, difficult‐to‐access anatomical location and highly desmoplastic, paucicellular nature, which limits the sensitivity of cytological and pathological diagnosis approaches.^4^ Considering the low rates of early detection, clinical silence and poor prognosis of CCA, clarifying the underlying mechanism and development of optional therapeutic targets for the sake of early detection, treatment and prognosis are crucial.

Aberrations in gene expression and DNA epigenetic alteration have been extensively studied for their roles in the pathogenesis and prognosis of many human cancers. An increasing number of studies have demonstrated crucial biological functions of epigenetic modifications, especially DNA methylation, in CCA, but there are limitations. Aberrant methylated cytosine in the CpG island of the promoter region of genes is one of the most remarkable characteristics of malignant cells and is believed to be an alternative mechanism underlying the transcriptional silencing of critical genes involved in carcinogenesis. Liu P et al.^5^ observed transcriptional inactivation and methylation of GATA5 in CCA tissues compared to noncancerous tissues, which was restored after treatment with 5‐aza‐2′‐deoxycytidine (5‐AZA). Upregulated GATA5 inhibited CCA cell growth and metastasis via the Wnt/β‐catenin pathway. Vedeld H. M. et al.^6^ analysed DNA methylation biomarkers in a set of bile samples, including those from patients with primary sclerosing choloangitis and CCA, using droplet digital PCR. They demonstrated that four DNA methylation biomarkers accurately differentiated patients with CCA from those with benign liver disease. The diagnostic accuracy for CCA was high in sporadic and PSC‐associated cancer, and DNA methylation markers were highly methylated in CCA tumour tissues. These results validate the utility of the methylation panel for early CCA diagnosis.

The liquid biopsy concept was introduced for circulating tumour cells (CTCs) a decade ago and has since been extended to circulating tumour DNA (ctDNA), circulating cell‐free DNA (cfDNA) and other tumour‐derived products.^7^ The term “liquid biopsy” refers to molecules such as proteins, DNA, RNA, tumour cells and other extracellular vesicles in blood and other bodily fluids that originate from the primary and metastatic tumour. A liquid biopsy provides a non‐invasive or minimally invasive method to extract genetic material from the tumour, with applications extending to early cancer detection, patient follow‐up, assessment of minimal residual disease and potential identification of molecular progression and the mechanism of resistance.^8^ Numerous liquid biopsy‐based studies have been conducted in human cancers, including lung, colorectal and breast cancers.^9^, ^10^, ^11^, ^12^, ^13^, ^14^, ^15^ Wasenang W et al.^16^ detected methylation of OPCML, HOXA9 and HOXD9 in the serum cell‐free DNA of patients with CCA and other biliary diseases using methylation‐sensitive high‐resolution melting and found significant differences in the methylation levels of OPCML and HOXD9.

Junctional adhesion molecule (JAM) is an immunoglobulin subfamily that has roles in tight junctions. The JAM subfamily contains six members, including JAM1–4, ESAM and CAR.^17^ The coding region of JAM3 is distributed over nine exons and maps to chromosome 11.q25.^18^ Numerous studies have demonstrated that JAM3 is an adhesion and transmigration regulatory element.^19^, ^20^, ^21^ More recently, hypermethylation of JAM3 has been reported in certain types of cancer.^17^, ^22^ However, the expression profile, methylation status, and function of JAM3 in CCA are unknown. In this study, we analysed the JAM3 expression level and methylation status in RBE, HCCC‐9810, HuCCT1 cell lines and CCA FFPE samples. Methylated JAM3 was detected in cfDNA from patients with CCA and other biliary diseases. Moreover, the biological roles of JAM3 were studied by transfection of JAM3 targeting siRNA and pCMV3‐JAM3 plasmid into CCA cell lines. The relationships between the expression and methylation status of JAM3 and the survival of patients with CCA were also analysed.

## 2 MATERIALS AND METHODS

### 2.1 CCA cell lines

The CCA cell lines RBE, HCCC‐9810 and HuCCT1 were purchased from the National Collection of Authenticated Cell Cultures of China. The cells were routinely cultured in RPMI‐1640 medium (KeyGEN BioTECH) supplemented with 10% foetal bovine serum (FBS; Gibco), penicillin (10 U/mL) and streptomycin (10 μg/mL) at 37°C, 95% air and 5% CO_2_.

### 2.2 Expression of JAM3 mRNA in CCA cell lines

Total RNA was extracted using the RNA extraction reagent TRIzol (Invitrogen) according to the manufacturer's protocol. Single strand cDNA was synthesized using 2 μg of total RNA with an oligo dT primer. RT‐PCR was performed to detect JAM3 transcript expression in the RBE, HCCC‐9810 and HuCCT1 cell lines. The primers for JAM3 were as follows: forward, 5′‐AGGTGTGAGGAGCAGGAGATGG‐3′; and reverse, 5′‐CCTCGTCAGTGCGGATGTAGTT‐3′. The PCR products were 211 bp, and the housekeeping gene GAPDH was used as an internal control. The primers for GAPDH were as follows: forward, 5′‐CGCAGGCCGGATGTGTTC‐3′; and reverse, 5′‐GGCGCCCAATACGACCAAA‐3′. The PCR products were 138 bp, and all were subjected to 3% agarose gel electrophoresis.

### 2.3 Restoration of JAM3 with 5‐AZA

RBE, HCCC‐9810 and HuCCT1 cells were treated with 5‐AZA (Sigma). Briefly, cells were cultured in 96 well plates overnight and treated with 5‐AZA (5 μM) dissolved in phosphate buffered saline (PBS) for 48 h.

### 2.4 Plasmid and siRNA transfection

Human pCMV3‐JAM3 plasmid with the full‐length open reading frame of the human JAM3 gene and pCMV3 vector were purchased from Sino Biological Inc. HCCC‐9810 and HuCCT1 cells with low expression of JAM3 were transfected with pCMV3‐JAM3 plasmid by Lipofectamine 2000 (Thermo Fisher Scientific Inc.) following the manufacturer's instructions. The pCMV3 vector was used as a control.

The sequences of small interfering RNA (siRNA) targeting JAM3 were synthesised as previously described.^17^ The sequences were JAM3 siRNA sense 5′‐UUCACUUGCACAGUUAACUCGAUCA‐3′ and antisense, 5′‐UGAUCGAGUUAACUGUGCAAGUGAA‐3′; nontargeting siRNA (siNC) control sense, 5′‐UUCUCCGAACGUGUCACGUTT‐3′ and control antisense 5′‐ACGUGACCGUUCGGAGAATT‐3′. siRNA synthesis was conducted by Sangon Biotech Company (Sangon Biotech). The transfection of a JAM3‐targeted siRNA into RBE cell with high expression of JAM3 was performed using Lipofectamine 2000 (Thermo Fisher Scientific Inc.) following the manufacturer's instructions.

### 2.5 Functional study of JAM3 on CCA cells

The proliferation, apoptosis, immigration and invasion tests were conducted after transfection of siRNA and plasmid. Firstly, the cells were transfected and cultured for 24 h until they reached 70% confluence. Next, MTT solution (Keygentec, Inc.) was added and incubated for 4 h. After adding dimethyl sulfoxide (DMSO) and shaking for 1 h, the absorbance was measured at 540 nm. Apoptosis was detected using an Annexin V‐FITC detection kit (Keygentec, Inc.). Cells were suspended in a binding buffer with 5 μL Annexin V‐FITC and 5 μL propidium iodide (PI). After incubation at room temperature for 10 min, annexin V‐FITC/PI binding was determined by flow cytometry. A wound healing assay was used to examine the effect of JAM3 on the invasive ability of CCA cells. After transfected cells had fully covered the plate, a wound was made with a sterile pipette tip, and the relative distance of the wound was photographed after 12 h. A Transwell insertion chamber was used for the migration assay according to the manufacturer's instructions.

### 2.6 Tumour tissues and blood samples

Sixty‐one patients with CCA who were surgically resected at the First Affiliated Hospital of Nanjing Medical University between January 2020 and December 2022 were studied. All samples were collected from patients without chemotherapy or other treatment before surgery. Formalin‐fixed and paraffin‐embedded tissue blocks (FFPE) of CCA were collected from the Department of Pathology of the First Affiliated Hospital of Nanjing Medical University. Peripheral blood samples were collected for extraction of cfDNA from 30 patients with CCA (10 iCCA, 8 dCCA and 2 pCCA) before therapeutic intervention and 30 patients with other biliary diseases (5 cholelithiasis and 15 cholangitis) as controls. The study protocol conformed to the ethical guidelines of the 2013 Declaration of Helsinki and was approved by the Ethics Committee of the First Affiliated Hospital of Nanjing Medical University (2019‐SR‐133). Written informed consent was obtained from all subjects.

### 2.7 Immunohistochemistry

The FFPE tissues were sectioned at 4‐μm thicknesses and then processed for immunohistochemical staining. Briefly, sections were deparaffinized and rehydrated, and endogenous peroxidase activity was blocked by incubation with 0.3% H_2_O_2_ for 10 min at room temperature. Antigen retrieval was performed by autoclaving the sections in 10 mM citrate buffer (pH 6.0) at 120°C for 20 min. The sections were incubated at 4°C overnight with a polyclonal anti‐JAM3 antibody (Invitrogen) diluted at 1:500. After washing, the sections were incubated with a secondary antibody (Dako REAL EnVision Detection System) for 30 min at room temperature. Finally, the colour was developed with 3,3′‐diaminobenzidine (DAB), and the nuclei were lightly counterstained with haematoxylin. Junctional adhesion molecular 3 expression was assessed for intensity as follows: 0 = no staining, 1 = weak staining, 2 = moderate staining and 3 = strong staining. Scores of 0 and 1 were defined as negative staining, while scores of 2 and 3 were defined as positive staining.

### 2.8 Methylated JAM3 in cell lines and FFPE tissues

#### 2.8.1 DNA extracted from cell lines

Total DNA was extracted from CCA cells using a FastPure Blood/Cell/Tissue/Bacteria DNA Isolation Mini Kit (NUOWEIZAN). In brief, 5 × 10^6^ cells were transferred to a sterile microcentrifuge tube adding 200 μL PBS, 20 μL proteinase, and 200 μL Buffer BC, before incubating at 56°C for 10 min. DNA was extracted using DNA purification mini‐columns.

#### 2.8.2 DNA extracted from FFPE

For each FFPE sample, 10‐μm thick sections were cut. Five sections were placed into 1.5 mL microcentrifuge tubes for DNA extraction, which was performed using the QIAmp DNA FFPE Tissue kit (Qiagen) according to the manufacturer's instructions. A NanoDrop spectrophotometer was used to quantify the DNA concentration and quality.

#### 2.8.3 Bisulfite modification of DNA

Bisulfite modification of genomic DNA was performed using the EZ DNA Methylation‐Gold Kit (Catalogue No. D5005; ZYMO Research Corp.) according to the manufacturer's instruction.

#### 2.8.4 Bisulfite sequencing PCR

Methylation detection in FFPE specimens and cell lines was conducted using a bisulfite sequencing PCR (BSP). The BSP primers were as follows: forward, 5′‐GTAGGGTTTTGGTAGGTTGGG‐3′; reverse, 5′‐CCCTAAAAAACAACAACAAAAAAAA‐3′. The PCR products were 159 bp, all of which were subjected to direct Sanger sequencing.

### 2.9 Methylated JAM3 was detected in cfDNA using qMSP

#### 2.9.1 Extraction of cfDNA

Peripheral blood was extracted from patients and maintained in cell‐free DNA blood collection tubes. cfDNA was extracted using the Quick‐cfDNA serum kit (ZYMO RESEARCH) according to the protocol provided by the manufacturer.

#### 2.9.2 Fluorescence‐based real‐time PCR technique (qMSP)

Considering that the amount of cfDNA is very small, hypermethylation analysis of JAM3 in cfDNA was subjected to a MethyLight technology, which is a sensitive and fluorescence‐based real‐time PCR technique (qMSP). The NCBI reference sequence of JAM3 is NC_000011.10. The primer for JAM3‐qMSP‐F was 5′‐GGACGTTCGGTAGTTGGATC‐3′, that for JAM3‐qMSP‐R was 5′‐CTAAAAAAAACCCACCCGAA‐3′, and the JAM3‐qMSP‐Probe was Rox‐5′‐AAACCCCGCCCGAAAAAAACCC‐3′‐BHQ2. The PCR products were 164 bp, and GAPDH was used as an internal control for unmethylated DNA and bisulfite modification. The NCBI reference sequence for GAPDH is NC_000012.12. The primer for GAPDH‐qMSP‐F was 5′‐TGGATATTGTTGTTATTAATGATT‐3′, that for GAPDH‐qMSP‐R was 5′‐AAATATAAAAAAACTACCCATCAACC‐3′, and the GAPDH‐qMSP‐Probe was VIC‐5′‐TCCTCCCACACCAACTTTAAAACTCACCA‐3′‐BHQ1. The PCR products were 124 bp. Evaluation of qMSP was assessed on Ct value of JAM3 and GAPDH. Briefly, (1) CtGAPDH ≥ 35, the result is uninterpreted; (2) CtGAPDH < 35, δCt (CtJAM3‐CtGAPDH) ≤ 10, the result is positive; (3) CtGAPDH < 35, δCt (CtJAM3‐CtGAPDH) > 10, the result is negative.

### 2.10 Gene set cancer analysis database analysis

The GSCA database was used to analyse the relationship between JAM3 expression and methylation and the survival of patients.

### 2.11 Statistical analysis

Statistical analysis was performed using SPSS software (SPSS version 13.0 for Windows, IBM). The relationship between the expression level of JAM3 and clinicopathological parameters was analysed by Spearman's rank correlation test. Kaplan–Meier curves associated with the log‐rank test were used to analyse survival. Statistical significance was set at *p* < 0.05.

## 3 RESULTS

### 3.1 Downregulation of JAM3 in CCA cell lines

**FIGURE 1:**
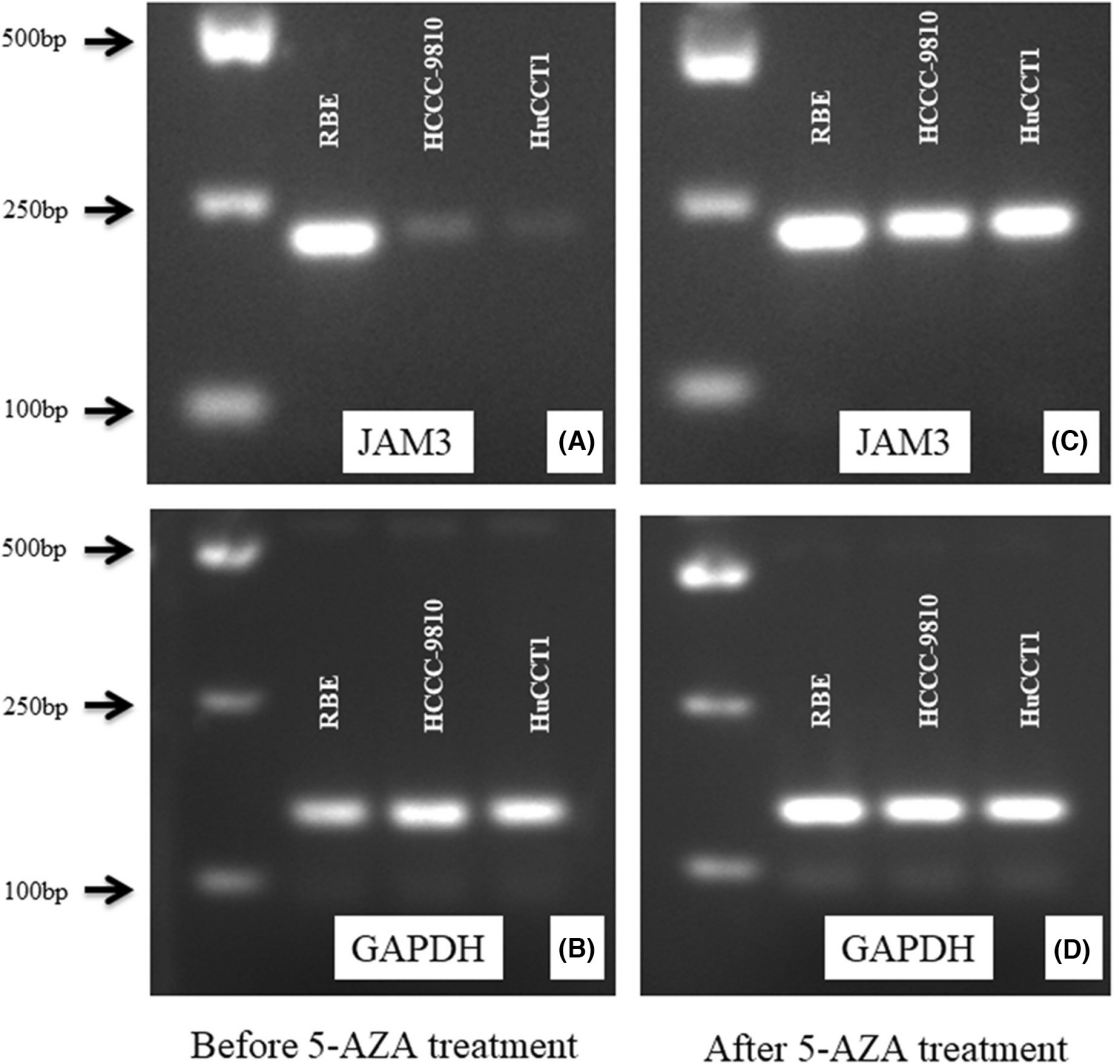
(A) Junctional adhesion molecular 3 mRNA high expressed in RBE, low expressed in HCCC‐9810 and lost in HuCCT1. (B) GAPDH used as an internal control. (C) JAM3 mRNA expression was restored by treatment of 5‐AZA in HCCC‐9810 and HuCCT1, no change in RBE. (D) GAPDH used as an internal control.

The expression of JAM3 mRNA in three cholangiocarcinoma cell lines, including RBE, HCCC‐9810 and HuCCT1 cells, was detected using RT‐PCR (Figure 1A). The housekeeping gene GAPDH was used as an internal control (Figure 1B). Junctional adhesion molecular 3 mRNA showed low expression in HCCC‐9810 and was lost in HuCCT1, but was highly expressed in RBE.

### 3.2 Methylation of JAM3 in CCA cell lines

**FIGURE 2:**
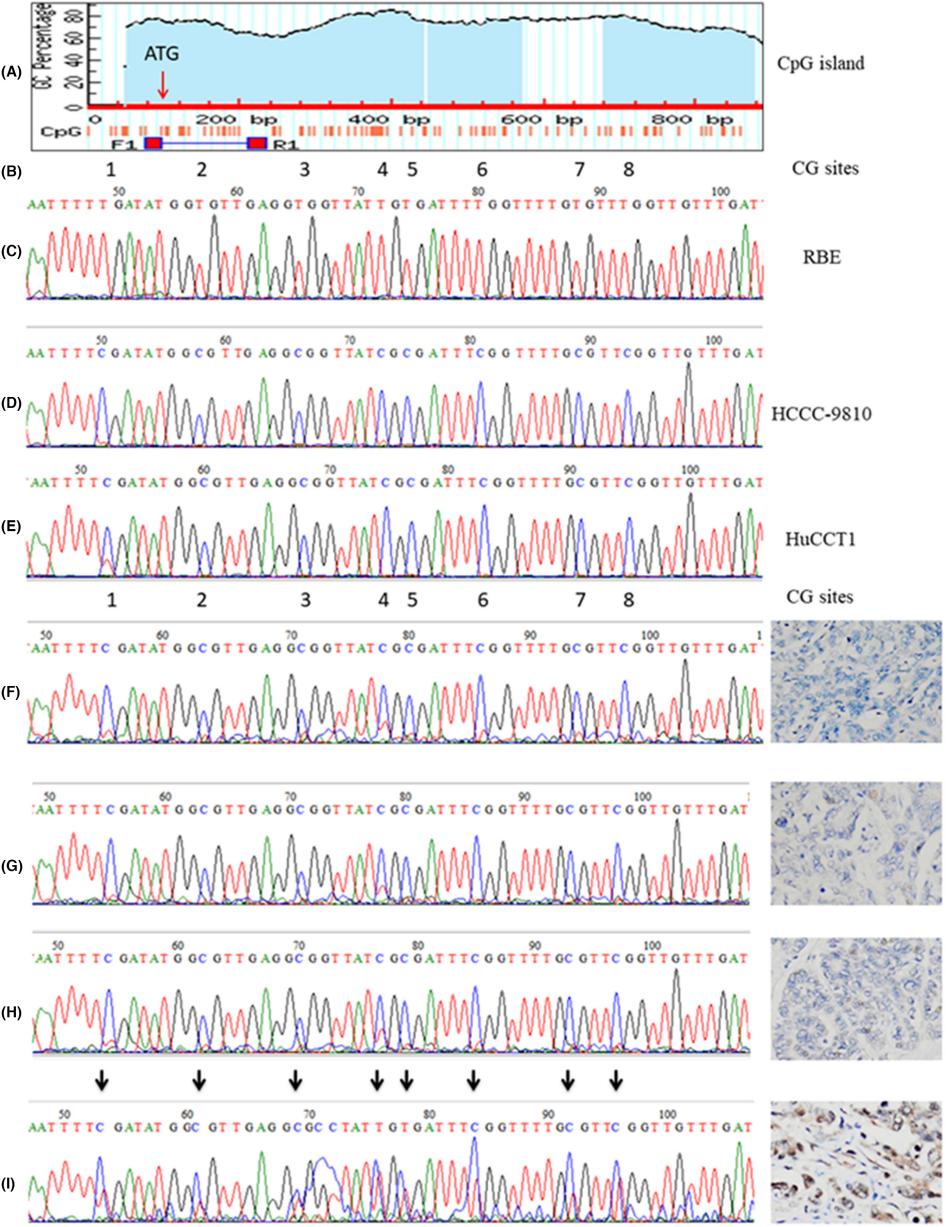
Bisulfite treated DNA direct sequencing (BSP) of RBE, HCCC‐9810, HuCCT1 CCA cell lines, and CCA tissues. (A) There are 3 CpG islands in the promoter related region of JAM3. BSP primer set was located around transcript start site (ATG). (B) There are 8 CG sites in the amplicon. (C) No methylated CG site was detected in RBE cells. (D, E) Methylated CG sites were detected in HCCC‐9810 and HuCCT1. Representative examples of BSP results correspond to immunohistochemical staining results of JAM3 in CCA tissues. (F, G, H) Methylated CG sites were detected in CCA tissues with negative staining of JAM3. (I) Both of methylated and unmethylated DNAs were detected in CCA tissue with positive staining of JAM3.

The methylation status of the CpG island in the region related to the JAM3 promoter (Figure 2A) in RBE, HCCC‐9810 and HuCCT1 cells was checked by bisulfite‐treated DNA direct sequencing (BSP).^23^ The hypermethylated CpG island in the region related to the promoter of JAM3 was detected in HCCC‐9810 and HuCCT1 cells but not in RBE cells (Figure 2B,C,D). The methylation status of CpG sites was significantly associated with the expression level of JAM3 mRNA.

### 3.3 5‐AZA rescued JAM3 expression

To further confirm the relationship between JAM3 expression and hypermethylation in CCA, three CCA cell lines were treated with 5‐AZA. As a result, the restoration of JAM3 mRNA was observed in HCCC‐9810 and HuCCT1 cells, but not in RBE (Figure 1C). The housekeeping gene GAPDH was used as an internal control (Figure 1D).

### 3.4 JAM3 functions as a tumour suppressor

**FIGURE 3:**
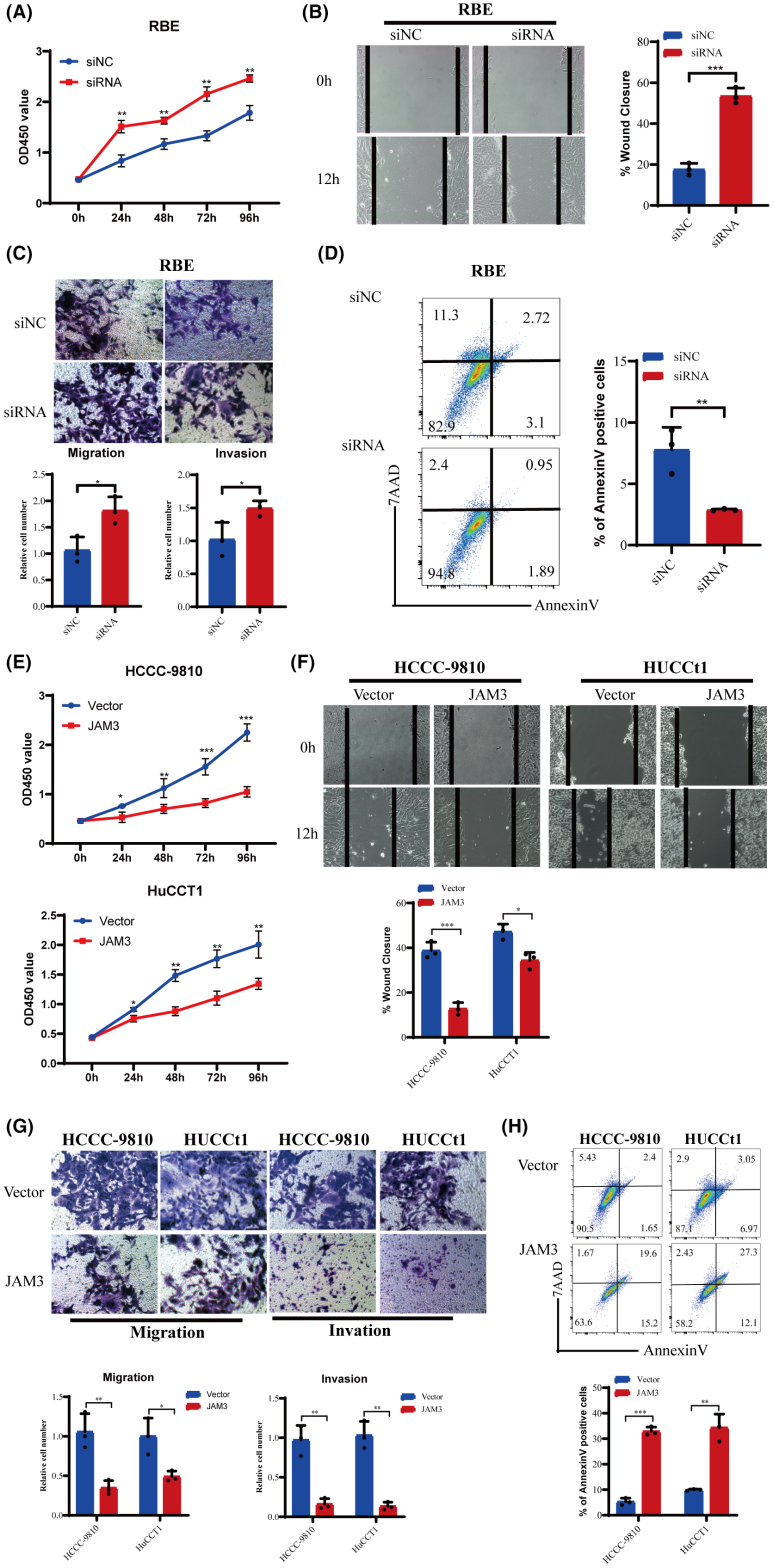
Biological function studies of JAM3 were carried out on RBE, HCCC‐9810 and HuCCT1 CCA cell lines with transfection of siRNA and pCMV3‐JAM3 plasmid. JAM3 depletion by transfection of targeting siRNA in RBE cells significantly increased the proliferation, invasion and migration and suppressed apoptosis (A–D). Transfection of pCMV3‐JAM3 plasmid significantly suppressed the proliferation, invasion and migration and induced apoptosis (3E–H) of HCCC‐9810 and HuCCT1 cells after transfected with pCMV3‐JAM3 plasmid compared to the corresponding control. (Magnification × 200). *p < 0.05, **p 0.01, ***p < 0.001.

The biological functions of JAM3 in RBE, CCC‐9810 and HuCCT1 cells were explored. The cellular proliferation, migration, invasion and apoptosis were compared in CCA cells with transfection of siRNA or plasmid to control. Junctional adhesion molecular 3 depletion by transfection of targeting siRNA in RBE cells significantly increased the proliferation, invasion and migration and suppressed apoptosis (Figure 3A–D). Transfection of pCMV3‐JAM3 plasmid significantly suppressed the proliferation, invasion and migration and induced apoptosis (Figure 3E–H) of HCCC‐9810 and HuCCT1 cells after transfected with pCMV3‐JAM3 plasmid compared to the corresponding control.

### 3.5 Loss of JAM3 protein in CCA tissues

**FIGURE 4:**
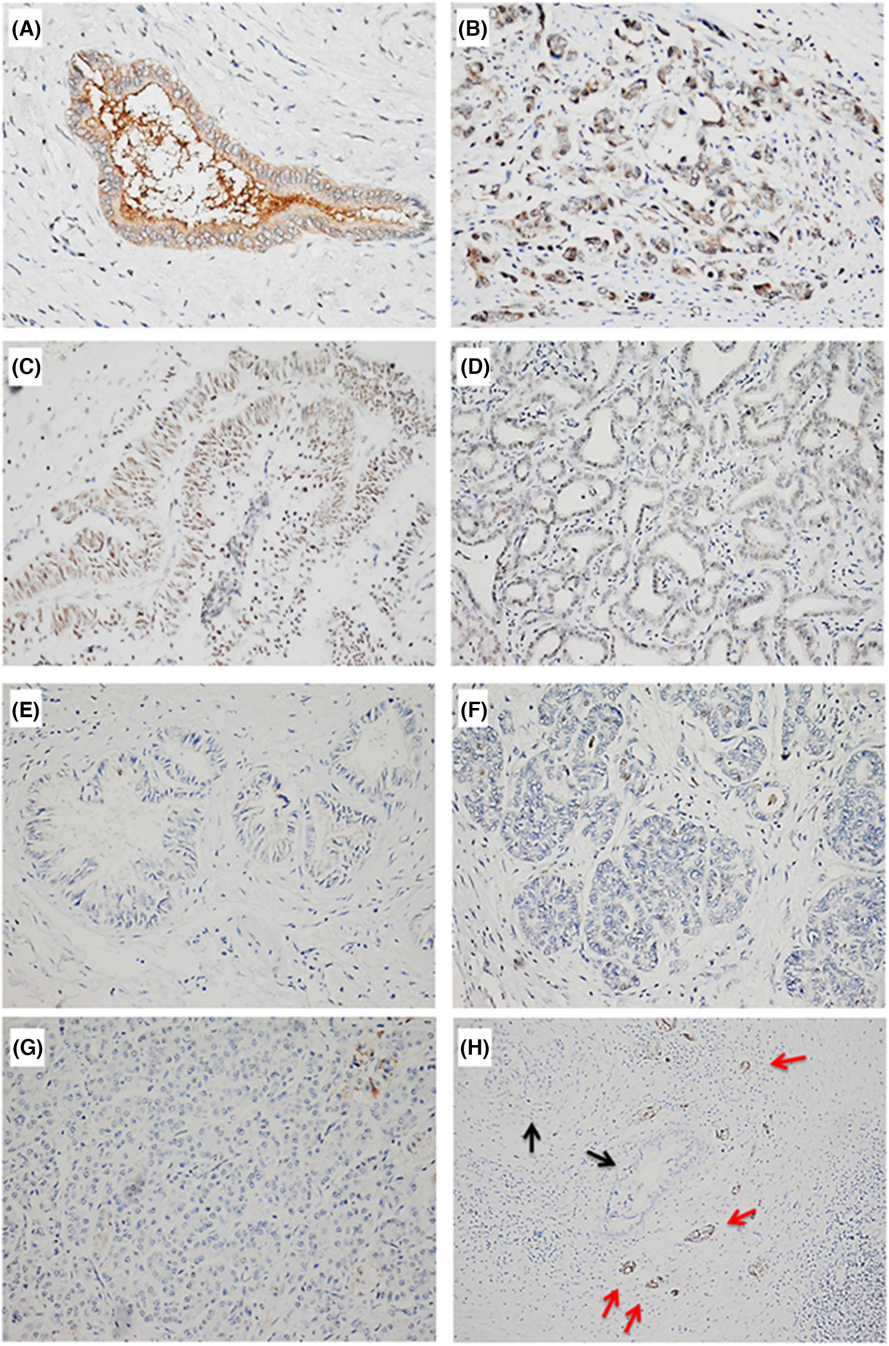
JAM3 protein was positive expressed in normal bile duct cells and differentially expressed in tumour cells of CCA. (A) JAM3 was positively expressed in normal bile duct cells. (magnification 400×) (B) JAM3 was positively expressed in tumour cells of CCA. (magnification 400×) (C) JAM3 was moderately expressed in tumour cells of CCA. (magnification 400×) (D) JAM3 was weakly expressed in tumour cells of CCA. (magnification 400×) (E, F, G) JAM3 was negatively expressed in tumour cells of CCA. (magnification 400×) (H) JAM3 was positively expressed normal bile duct cells (red arrow) and negatively expressed in tumour cells of CCA (black arrow). (magnification 100×).

The expression level of JAM3 protein in CCA tissues was checked using immunohistochemical staining with a specific antibody for JAM3. The JAM3 protein was located in the cytoplasm, as identified by homogeneous yellow staining, and positively expressed in normal bile duct cells (Figure 4A), but low or negatively expressed in most CCA tumour cells (Figure 4B–H). As shown in Table 1, JAM3 protein was negatively expressed in 44 out of 61 CCA specimens (72.1%) and positively detected in 17 out of 61 CCA specimens (27.9%). The relationship between JAM3 expression and clinicopathological parameters was statistically analysed, and no significant relationship between JAM3 expression and clinicopathological parameters was found.

**TABLE 1:** Association of JAM3 expression and hypermethylation with clinicopathological parameters.

	No	JAM3 protein		*p‐*Value	JAM3 qMSP		*p‐*Value
		−	+		U	M	
Age		44	17		16	45	
<65	42	29	13	0.433	14	28	0.062
≥65	19	15	4		2	17	
Gender							
Female	25	20	5	0.615	5	20	0.365
Male	36	24	12		11	25	
Subtypes							
pCCA	11	8	3	0.717	3	8	
dCCA	13	7	6		6	7	0.299
iCCA	37	29	8		7	30	
Differentiation							
Poor	23	21	2	0.252	2	21	0.011
Moderate	32	20	12		11	21	
Well	6	3	3		3	3	
Metastasis							
No	45	29	16	0.322	15	30	0.035
Yes	16	15	1		1	15	
TNM stage							
I + II	30	14	16	0.053	15	15	<0.001
III + IV	31	30	1		1	30	

### 3.6 JAM3 was frequently methylated in CCA tissues

The methylation status at the CpG island in the promoter‐related region of JAM3 in CCA specimens was analysed by BSP assay following the treatment of genomic DNA with sodium bisulfite. As shown in Table 1, methylated JAM3 DNA was detected in 45 out of 61 CCA specimens (73.8%), and no methylation was detected in 16 out of 61 CCA specimens (26.2%). The methylation status of CpG sites was significantly related to the JAM3 protein expression level (Figure 3). Methylation of JAM3 was associated with tumour differentiation (*p* = 0.011), metastasis (*p* = 0.035) and TNM stage (*p* < 0.001).

### 3.7 Detection of JAM3 hypermethylation in cfDNA samples

Methylated JAM3 was detected in cfDNA from CCA and biliary diseases using high‐throughput qMSP (Table 2). Methylated JAM3 was positively detected in 1 out of 2 iCCA, 3 out of 8 dCCA and 12 out of 20 pCCA. The sensitivity of JAM3 methylation detection was 53.3% (16/30). Methylated JAM3 was also detected in 1 out of 15 cholangitis samples and was not detected in 5 cholelithiasis samples. The specificity of JAM3 methylation detection was 96.6% (29/30). Methylation of JAM3 DNA was significantly higher in CCA compared to biliary diseases (*p* < 0.001).

**TABLE 2:** Methylation of JAM3 in cfDNA of CCA and biliary diseases.

	No	Methylation of JAM3 in cfDNA		*p‐*Value
		positive	negative	
CCA				
iCCA	2	1	1	<0.001
dCCA	8	3	5	
pCCA	20	12	8	
Biliary disease				
Cholelithiasis	5	0	5	
Cholangitis	15	1	14	

### 3.8 Relationship between JAM3 expression and hypermethylation and survival

**FIGURE 5:**
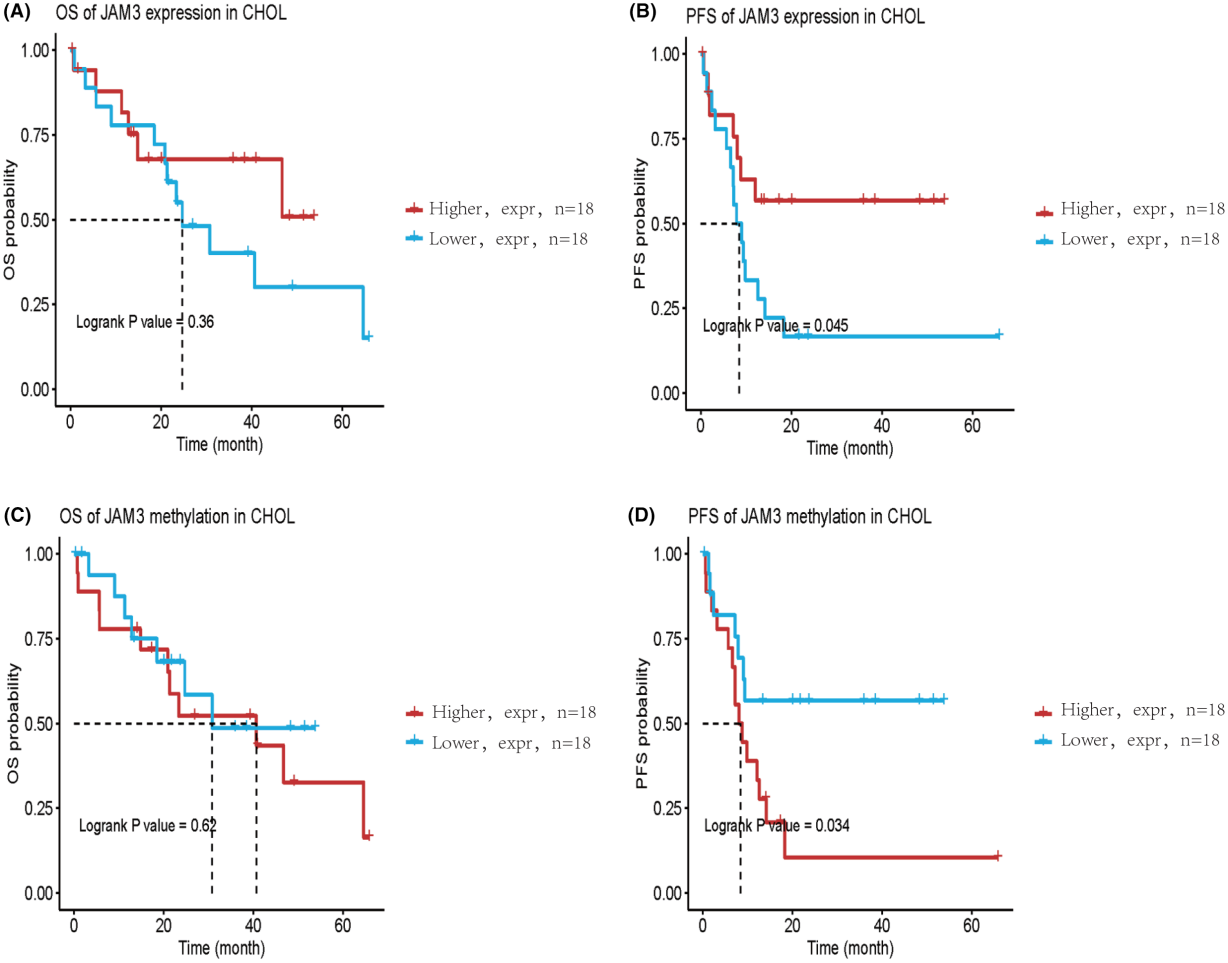
Survival analysis based on GSCA (Gene Set Cancer Analysis) database. (A) No significant difference was found between low and high expression of JAM3 in CCA patient's overall survival (*p* = 0.36). (B) Progression‐Free Survival (PFS) was significantly longer in patients with JAM3 high expression (*p* = 0.045). (C) No difference in overall survival for patients in methylation status (*p* = 0.62). (D) CCA patients with hypermethylation of JAM3 had a poor progression‐free survival than those with unmethylated (*p* = 0.034).

In this study, we used the GSCA database to investigate the relationship between JAM3 mRNA expression and hypermethylation and CCA patient survival (Figure 5). As a result, 18 cases of high JAM3 expression and 18 cases of low JAM3 expression were identified and followed up for survival analysis. The results showed no significant difference in overall survival between patients with low and high expression of JAM3 (*p* = 0.36). The progression‐free survival (PFS) was significantly longer in patients with high JAM3 expression (*p* = 0.045). Patients with CCA with JAM3 hypermethylation had a poorer PFS than those with unmethylated JAM3 (*p* = 0.034), but no significant difference in overall survival (*p* = 0.62).

## 4 DISCUSSION

The JAM family shows both distinct and overlapping patterns of tissue expression. JAM1, 2 and 3 are expressed in the placenta, and JAM3 is expressed in the brain, kidney, and T cells.^18^ The expression profile, role and mechanism of JAM3 vary according to cancer type, and in some cases, are even paradoxical. Junctional adhesion molecular 3 is positively expressed in HK2, Caki1 and 7860 renal carcinoma cell lines. The exogenous knockdown of JAM3 has been shown to inhibit renal carcinoma cell migration and promote renal carcinoma cell apoptosis via regulation of E‐cadherin, N‐cadherin, integrin β1 and MMP2 expression.^24^ Leukaemia‐initiating cells, which present high JAM3 expression, are important for the initiation, development, and relapse of leukaemia, while knockdown of JAM3 has been shown to dramatically decrease the proliferation of leukaemia cell lines and primary leukaemia‐initiating cells.^25^ Junctional adhesion molecular 3 has been shown to be downregulated by hypermethylation in cervical cancer, colorectal cancer, and oesophageal cancer.^17^, ^22^, ^23^ Moreover, JAM3 methylation status in cervical tissues and scraping demonstrated a significant difference between low‐ and high‐grade cervical intraepithelial neoplasia (CIN) and normal cervical tissue and CIN, suggesting that JAM3 methylation could be a biomarker for the diagnosis of cervical cancer and for screening.^22^ Junctional adhesion molecular 3 has also been shown to be frequently methylated and downregulated in colorectal cancer tissues, cell lines and plasma samples, while restoration of JAM3 has been found to repress CRC cell viability, colony formation and migration. Furthermore, knockout of JAM3 in NCM460 cells has been demonstrated to improve clonogenicity and migration capability, as well as suppress apoptosis and cell‐cycle arrest.^17^ Junctional adhesion molecular 3 shows differential expression in oesophageal cancer cell lines, with an inverse association observed between JAM3 RNA expression and methylation. Junctional adhesion molecular 3 also suppresses cell proliferation, colony formation, migration and invasion and induces cell cycle arrest and apoptosis via Wnt/β‐catenin signalling. The methylation of JAM3 is an independent prognostic factor of overall survival for oesophageal cancer.^23^

Although the expression of JAM3 has been reported previously in different human cancers, its biological role remains controversial. To the best of our knowledge, the expression profile, methylation status and biological role of JAM3 in CCA are unknown. In the present study, we reported that JAM3 mRNA expression was low in HCCC‐9810, lost in HuCCT1 and high in RBE cells, while JAM3 protein expression was either low or lost in CCA tissues. Hypermethylation of JAM3 was frequently observed in CCA tissues, and the methylation status of CpG island sites was significantly related to the expression level of JAM3 mRNA or protein in CCA cell lines and tumour tissues. Methylation of JAM3 was also found to be associated with tumour differentiation, metastasis and TNM stage, while transfection of JAM3 induced apoptosis and repressed CCA cell viability, invasion and migration. Our data suggest that JAM3 serves as a tumour suppressor in CCA. To the best of our knowledge, this is the first study to report the expression, methylation status and biological function of JAM3 in CCA.

Diagnosis of CCA is based on a combination of clinical, radiological, biochemical and histological approaches, and most patients have advanced‐stage disease at presentation.^4^, ^26^ Diagnosis of CCA at an early stage is challenging because of its asymptomatic clinical manifestation, difficult access to anatomical location and lack of a sensitive and specific biochemical assay. Furthermore, the highly desmoplastic and paucicellular nature of CCA limits the sensitivity of cytological and pathological diagnosis.^4^ Therefore, extensive research is necessary to identify biomarkers that can be used to assist in the early diagnosis of CCA. Hypermethylation of tumour suppressor genes plays an important role in tumourigenesis and tumour progression and may represent an early molecular event in malignant transformation.^27^, ^28^, ^29^, ^30^ Junctional adhesion molecular 3 has been shown to participate in epithelial‐mesenchymal transition (EMT) in colonic epithelial cells and functions as a tumour suppressor in colorectal cancer.^17^ Aberrant methylation patterns in ctDNA have been implicated as useful tools for non‐invasive cancer detection.^31^, ^32^, ^33^, ^34^, ^35^ We detected methylated JAM3 in cfDNA from patients with CCA and biliary diseases, with a sensitivity of 53.3% and specificity of 96.6%. Our data suggested that JAM3 serves as a potential biomarker for the diagnosis or early detection of CCA.

Given the poor prognosis of CCA, especially iCCA, efforts have recently focused on improving prognostication and optimising selection criteria to identify the best candidates for surgery.^36^, ^37^, ^38^, ^39^ Traditional predictors of outcomes among patients with iCCA include tumour burden, differentiation, grade, microvascular invasion, lymph node metastasis and tumour markers (CA).^40^, ^41^ However, so far, available prognostic factors have not accurately predicted the prognoses of patients with iCCA. In this study, we found that the PFS of patients with CCA was significantly longer in patients with high JAM3 expression and hypermethylation. Our data indicate that JAM3 expression and hypermethylation may be used as a new candidate factor to predict the prognosis of CCA.

## 5 CONCLUSIONS

Junctional adhesion molecular 3 was frequently downregulated in CCA due to its hypermethylation. Methylated JAM3 could be detected in cfDNA with high specificity and moderate sensitivity. High JAM3 expression and unmethylated JAM3 DNA are favourite survival predictors for patients with CCA, suggesting that JAM3 may be a non‐invasive biomarker for the diagnosis and prognosis of CCA.

## AUTHOR CONTRIBUTIONS

**Yi Shi:** Conceptualization (equal); data curation (equal). **Xiao Feng:** Investigation (equal); methodology (equal). **Ying Zhang:** Data curation (equal). **Ji Gao:** Investigation (equal); methodology (equal). **Wei Bao:** Funding acquisition (equal); investigation (equal). **Jian‐dong Wang:** Project administration (lead); writing – review and editing (lead). **Jian‐feng Bai:** Formal analysis (equal); investigation (equal).

## CONFLICT OF INTEREST STATEMENT

The authors declare no competing interest.

## Data Availability

The data that support the findings of this study are available from the corresponding author upon reasonable request.
